# Association of a novel frameshift variant and a known deleterious variant in *MMR* genes with Lynch syndrome in Chinese families

**DOI:** 10.1186/s12957-024-03309-5

**Published:** 2024-01-27

**Authors:** Juyi Li, Haichun Ni, Xiufang Wang, Wenzhuo Cheng, Li Li, Yong Cheng, Chao Liu, Yuanyuan Li, Aiping Deng

**Affiliations:** 1 Department of Pharmacy, The Central Hospital of Wuhan, Tongji Medical College, Huazhong University of Science and Technology, Wuhan, Hubei China; 2 Department of Pathology, The Central Hospital of Wuhan, Tongji Medical College, Huazhong University of Science and Technology, Wuhan, Hubei China; 3 Department of Pain, The Central Hospital of Wuhan, Tongji Medical College, Huazhong University of Science and Technology, Wuhan, Hubei China; 4 Department of Endocrinology, Institute of Geriatric Medicine, Liyuan Hospital, Tongji Medical College, Huazhong University of Science and Technology, Wuhan, Hubei China; 5 Department of Oncology, The Central Hospital of Wuhan, Tongji Medical College, Huazhong University of Science and Technology, Wuhan, China; 6 Department of Gastrointestinal Surgery, The Central Hospital of Wuhan, Tongji Medical College, Huazhong University of Science and Technology, Wuhan, Hubei China; 7 Hubei Key Laboratory of Diabetes and Angiopathy, Hubei University of Science and Technology, Xianning, Hubei 437000 China

## Abstract

**Background:**

Lynch syndrome (LS) is the most common hereditary colorectal cancer (CRC) syndrome. This condition is characterized by germline variants in DNA mismatch repair (MMR) genes, including *MLH1*, *MSH2*, *MSH6*, and *PMS2*. In this study, we analyzed the molecular defects and clinical manifestations of two families affected with CRC and proposed appropriate individual preventive strategies for all carriers of the variant.

**Methods:**

We recruited two families diagnosed with CRC and combined their family history and immunohistochemical results to analyze the variants of probands and those of other family members by using whole exome sequencing. Subsequently, gene variants in each family were screened by comparing them with the variants available in the public database. Sanger sequencing was performed to verify the variant sites. An online platform (https://www.uniprot.org) was used to analyze the functional domains of mutant proteins.

**Results:**

A novel frameshift variant (NM_001281492, c.1129_1130del, p.R377fs) in *MSH6* and a known deleterious variant (NM_000249.4:c.1731G > A, p.S577S) in *MLH1* were identified in the two families with CRC. Using bioinformatics tools, we noted that the frameshift variant reduced the number of amino acids in the MSH6 protein from 1230 to 383, thereby leading to no MSH6 protein expression. The silent variant caused splicing defects and was strongly associated with LS. 5-Fluorouracil-based adjuvant chemotherapy is not recommended for patients with LS.

**Conclusions:**

The novel frameshift variant (*MSH6*, c.1129_1130del, p.R377fs) is likely pathogenic to LS, and the variant (*MLH1*, c.1731G > A, p.S577S) has been further confirmed to be pathogenic to LS. Our findings underscore the significance of genetic testing for LS and recommend that genetic consultation and regular follow-ups be conducted to guide individualized treatment for cancer-afflicted families, especially those with a deficiency in *MMR* expression.

## Background

Colorectal cancer (CRC) is among the most common malignant tumors of the gastrointestinal system and includes both colon and rectal cancers [1–4]. Lynch syndrome (LS), also known as hereditary nonpolyposis CRC, is an autosomal dominant genetic disease, accounting for approximately 1–3% of all CRCs [5], with a population frequency of 1 in 440 individuals [6]. Patients with LS are more likely to develop cancers of the stomach, small intestine, and brain. Female patients with LS are also at risk of developing endometrial (25–60%) and ovarian cancers (4–12%) [7–9]. While diagnosing LS based on clinical manifestations is difficult, the average age of patients with LS tends to be lower [10, 11].

LS is primarily associated with deleterious germline variants in DNA mismatch repair (MMR) genes, including *MLH1*, *MSH2*, *MSH6*, and *PMS2* [12–15], as well as deletions in *EpCAM*. These variants and deletions cause LS through transcriptional silencing of the downstream *MSH2* gene and not due to the role of EpCAM in MMR [16]. A mutation in *MMR* alters MMR protein expression in patients with LS. As a result, the efficiency of MMR activity reduces, and cancer is induced through functional copy loss. When DNA replication errors cannot be corrected in time, the length of DNA microsatellite repeats changes, that is, microsatellite instability (MSI) occurs. MSI accelerates the accumulation of somatic variants, leading to tumor occurrence [17].

Early diagnosis, early intervention, and continuous monitoring are beneficial for prolonging the survival of patients with LS. Therefore, the LS-associated variant profile must be studied in the Chinese population. This would allow us to implement genetic counseling based on genetic testing. With the recent advances in sequencing technology, whole exome sequencing (WES) is gradually being used in clinical settings for identifying changes in all functional gene sequences in LS and other diseases [18–20]. This greatly improves the detection of LS and guides the selection of medication and treatment strategies for it [21].

Using WES and Sanger sequencing, we report a novel variant in *MSH6* and a known *MLH1* variant in two Chinese families diagnosed with LS. This study analyzed the molecular defects and clinical manifestations of these two families to provide appropriate individual preventive strategies for all identified variant carriers.

## Materials and methods

### Participants

The Ethics Committee of the Central Hospital of Wuhan approved this study, and informed consent was obtained from all participants. Families with CRC were recruited from the Central Hospital of Wuhan, and the primary affected members (probands) of each family underwent partial colon resection. LS was clinically diagnosed by oncologists based on a combination of the Amsterdam II criteria [22] and a detailed analysis of the family pedigree.

### Participants’ clinical characteristics

**Fig. 1:**
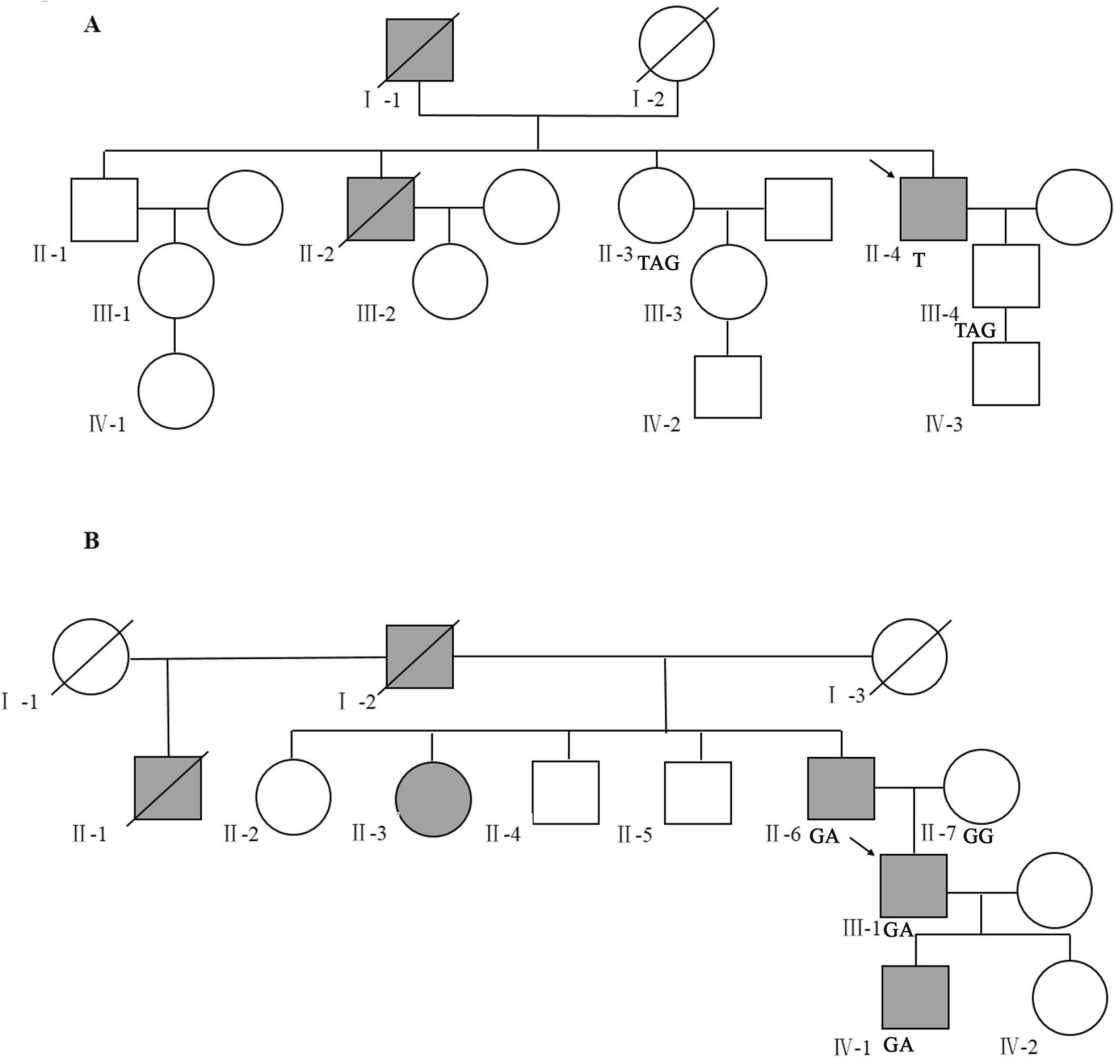
Family pedigree. Pedigrees of family 1 (**A**) and family 2 (**B**). Squares represent males and circles represent females. The proband is marked with an arrow. The gray squares indicate colorectal cancer, gastric cancer, or endometrial cancer, and IV-1 is a carrier of pathogenic mutations (family 2). Slash symbols indicate the deceased members

The proband (II-4) in family 1 was a 64-year-old man who had undergone a partial colon resection at 59 years because of adenocarcinoma of the descending colon. His father (I-1) and older brother (II-2) had both died of CRC at the ages of 62 and 66, respectively. His sister (II-3) and son (III-4) were in good health. Figure 1A provides the detailed pedigree. Three individuals (II-3, II-4, and III-4) from this family were included in this study.

The proband (III-1) of family 2 was a 30-year-old man who had been diagnosed with highly moderately differentiated tubular adenocarcinoma of the rectum and had undergone anterior rectal resection. At 57 years, the father of the proband (II-6) was diagnosed with poorly differentiated adenocarcinoma of the whole stomach and had undergone subtotal gastrectomy with gastrojejunostomy. Additionally, his grandfather (I-2) and his aunt (II-3) had CRC and endometrial cancer, respectively. One of his uncles (II-1) had died in his 20 s, whereas his mother (II-7) was in good health. Figure 1B presents the detailed pedigree. The proband’s father was operated on in a different hospital, and the postoperative pathological results revealed poorly differentiated adenocarcinoma of the whole stomach with focal signet ring cell carcinoma and lymph node metastasis. Four individuals (II-6, II-7, III-1, and IV-1) from this family were included in this study.

### Histological analysis

MMR expression was detected using previous methods [8]. Briefly, paraffin sections were stained with anti-MLH1, anti-PMS2, anti-MSH2, and anti-MSH6 antibodies.

### MSI test

The MSI test was performed as previously described [23]. Multiplex PCR analysis was performed to monitor the BAT-25, BAT-26, MONO-27, CAT-25, and NR-24 loci.

### DNA extraction and WES

Genomic DNA was extracted from the peripheral blood of all participants by using a commercial DNA extraction kit (TIANGEN, Beijing, China), following the manufacturer’s instructions. The obtained DNA was prepared for next-generation sequencing by using a commercial kit (SureSelect Human All Exon V5, Agilent), following the manufacturer’s protocol. Finally, exome sequencing was performed on the Illumina HiSeq2500 system.

### Sequence analysis

The sequenced paired reads were mapped to the NCBI Build 37 (hg19) reference genome. Single-nucleotide variants and indels were analyzed using public databases including GnomAD, ExAC, dbSNP, ESP, and 1000 Genomes, prioritizing LS-related variant genes cited in PubMed. Non-exonic, non-splicing, and synonymous variants were excluded, leaving only LS-associated variants for analysis. Additionally, in silico prediction tools such as SIFT, PolyPhen2, Mutation Taster, Mutation Assessor, FATHMM, and GERP plus were used to evaluate the relationships between the predicted pathogenic variants and LS.

### Sanger sequencing

Sanger sequencing was performed on the genomic DNA isolated from the peripheral blood. The primers used in PCR amplification were as follows: Forward (MLH1): 5′- CCACAGCCAGGCAGAACTATT-3′, Reverse (MLH1): 5′- GTTTAAGTTGGCTACCAAATGACTA-3′, exon 15 was amplified in *MLH1*; Forward (MSH6): 5′- TCCTGGGATGAGGAAGTGGT-3′, Reverse (MSH6): 5′- AGCACACACCATATGCACGA-3′; partial exon 4 was amplified in *MSH6*. The PCR reaction conditions were as follows: initial denaturation at 95 °C for 5 min; 35 cycles of denaturation at 95 °C for 1 min, annealing at 60 °C for 30 s, and extension at 72 °C for 1 min; followed by a final extension at 72 °C for 10 min.

### Analysis of the functional domains of mutant proteins

The effects of the variant on the MSH6 protein were analyzed by comparing it with the wide-type protein [UniProtKB-P52701 (MSH6_HUMAN), https://www.uniprot.org/uniprot/P52701/protvista]. Similarly, the variant in the MLH1 protein was analyzed in comparison to its wild-type counterpart, UniProtKB-P40692 (MLH1_HUMAN) (https://www.uniprot.org/uniprot/P40692/protvista).

## Results

### Immunohistochemical analysis

**Fig. 2:**
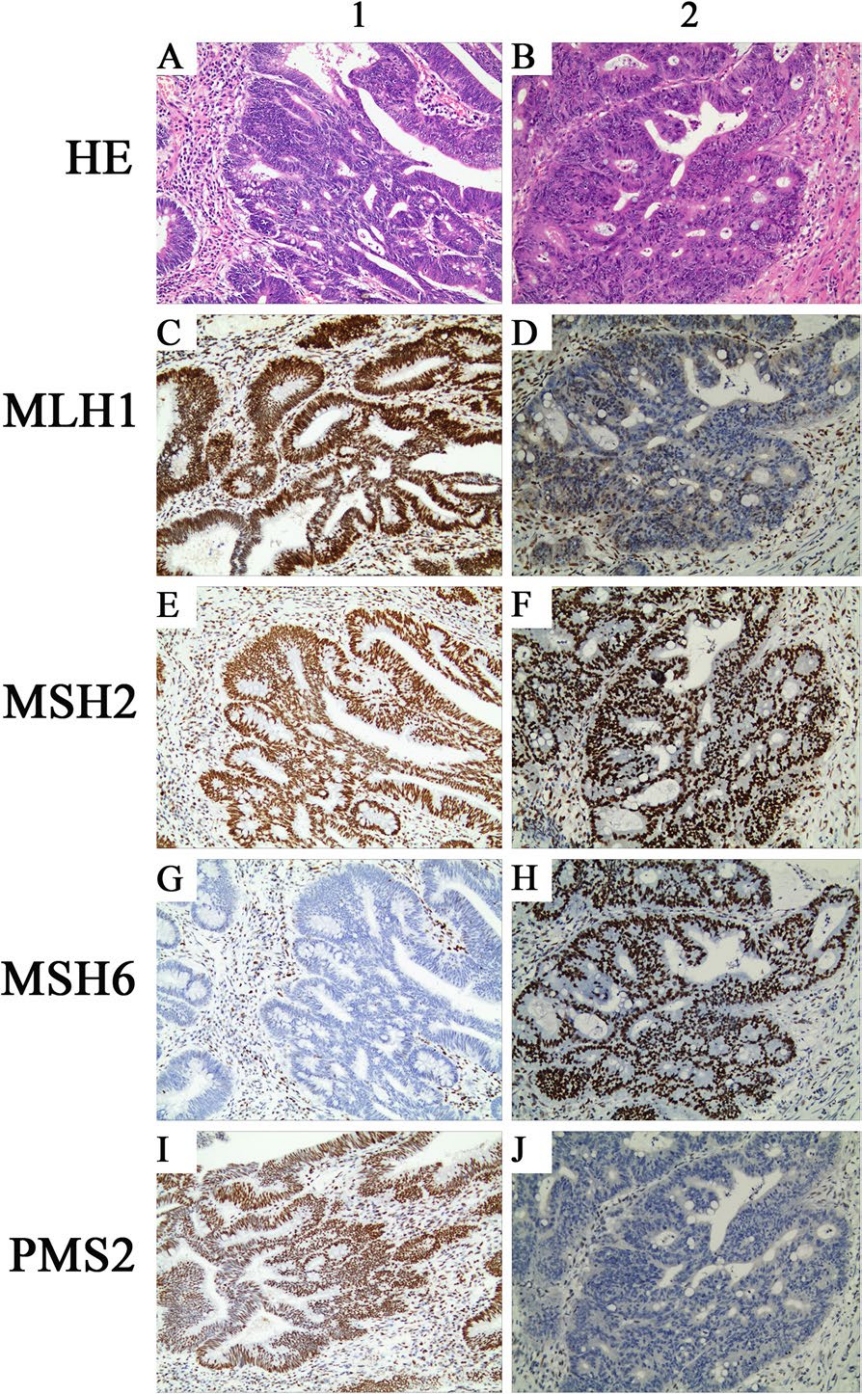
MMR protein expression of the probands in the two families. **A**, **B** Hematoxylin–eosin staining of the proband’s tumor tissues. Histopathological examination of the tumor revealed villous-tubular adenoma accompanied by high-grade glandular intraepithelial neoplasia and focal sections of moderately and well-differentiated intramucosal adenocarcinoma. **C**–**J** Immunohistochemistry analysis. From the left to right, images show staining of the proband’s tumor tissues from families 1 and 2, respectively. From top to bottom, the antibodies in each line are specific for MLH1, MSH2, MSH6, and PMS2. Loss of expression of MSH6 in family 1 and loss of expression of MLH1/PMS2 in family 2 were observed. All images were captured at 200 × magnification

Immunohistochemical staining of the proband’s tumor cells in family 1 demonstrated strong positivity for MLH1 (Fig. 2C), MSH2 (Fig. 2E), and PMS2 (Fig. 2I) proteins, and no staining for the MSH6 (Fig. 2G) protein. Immunohistochemical staining of the proband’s tumor cells in family 2 revealed strong positivity for MSH2 (Fig. 2F) and MSH6 (Fig. 2H) proteins and weak positivity for MLH1 (Fig. 2D) and PMS2 (Fig. 2J) proteins.

### Microsatellite analysis

For the proband in family 2, PCR analysis was performed on both rectal cancer tissue samples and normal tissue samples at five microsatellite repeat loci: BAT-25, MONO-27, CAT-25, BAT-26, and NR-24. All these markers showed a leftward shift in the cancer tissues compared to the normal tissues. This indicated that all five monomorphic mononucleotide markers were altered, and the results confirmed the presence of high MSI (MSI-H). For the proband in family 1, no results were obtained from the MSI test because of the patient’s inability to pay the pathological testing fees.

### Variant detection

By using WES, all MMR variants were detected and a likely pathogenic variant in *MSH6* was identified (family 1). Table 1 summarizes the WES data of the proband (II-4) in family 1.

**Table 1:** Whole exome sequencing detail of the probands in two families

Exome capture statistics	Proband in family 1	Proband in family 2
Total (bp)	69,442,952 (100.00%)	69,799,104 (100%)
Duplicate (bp)	14,862,727 (21.40%)	15,433,387 (22.11%)
Mapped (bp)	69,375,924 (99.90%)	69,745,856 (99.92%)
Properly mapped (bp)	68,415,120 (98.52%)	68,695,794 (98.42%)
PE mapped (bp)	69,315,566 (99.82%)	69,703,330 (99.86%)
SE mapped (bp)	120,716 (0.17%)	85,052 (0.12%)
Initial bases on target (bp)	60,456,963	60,456,963
Initial bases on or near target (bp)	136,297,444	136,297,444
Total effective yield (Mb)	10,368.49	10,423.95
Effective yield on target (Mb)	7307	7573.99
Fraction of effective bases on target (%)	70.47	72.66
Fraction of effective bases on or near target (%)	89.99	92.24
Average sequencing depth on target	121	125
Bases covered on target (bp)	60,260,584	60,309,703
Coverage of target region (%)	99.68	99.76
Fraction of target covered with at least 100 × (%)	50.75	55.75
Fraction of target covered with at least 50 × (%)	78.36	84.33
Fraction of target covered with at least 10 × (%)	97.28	98.37
Total SNPs	106,065	106,039
Novel SNPs	653	654
Total InDels	11,478	11,168
Novel InDels	765	704
Gender	Male	Male

**Fig. 3:**
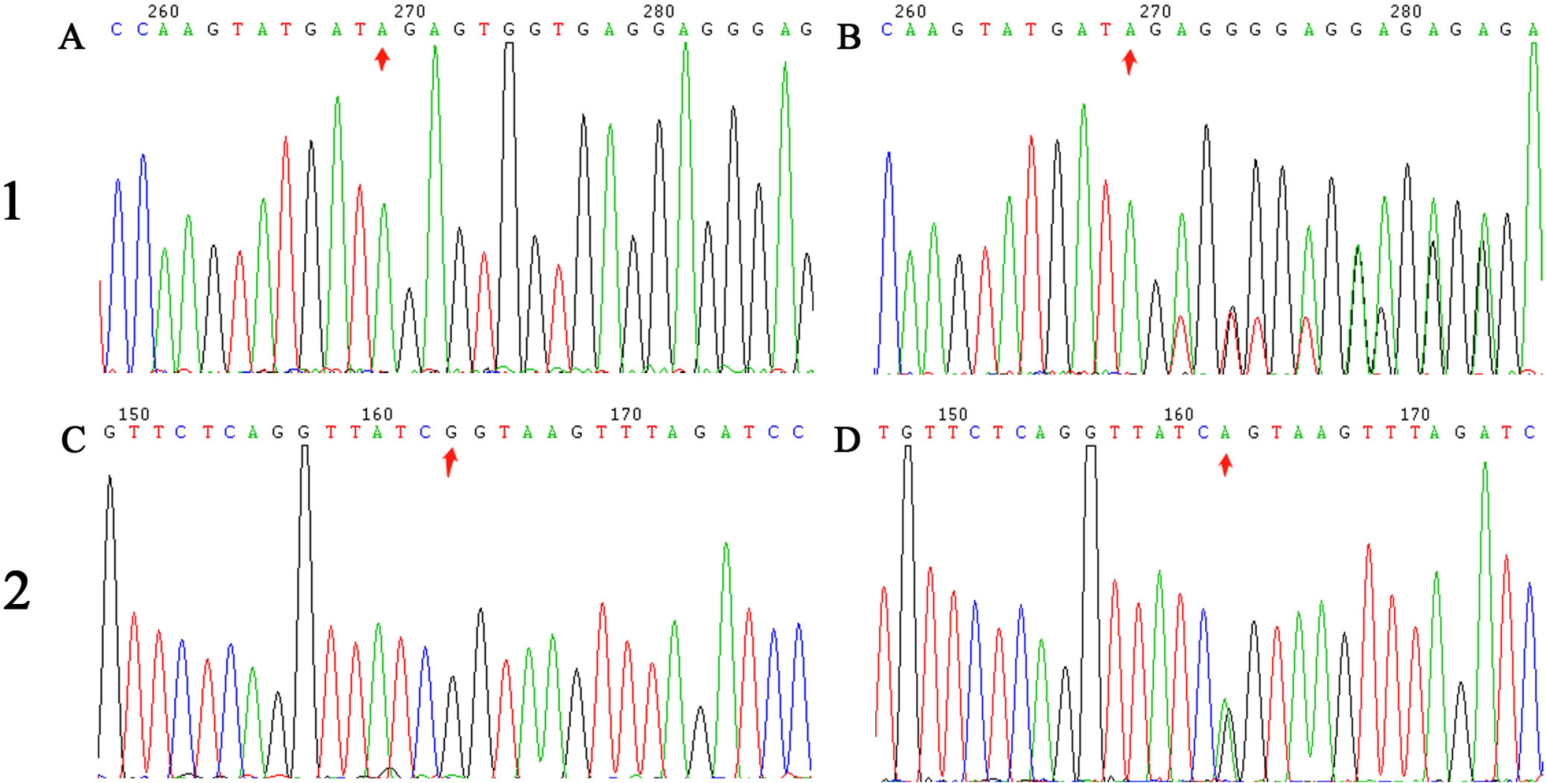
The MMR loci sequencing data. (1) Sanger sequencing analysis of *MSH6* gene (c.1129_1130del, p.R377fs) of the proband in family 1. **A** Wild type, **B** Mutant type, two bases were missing in exon 2. (2) Sequencing analysis of *MLH1* gene (c.1731G > A, p.S577S) of the proband in family 2. **C **Wild type, **D** Mutant type, a synonymous mutation in exon 15 were identified

In the proband, a novel frameshift variant was identified in *MSH6* (MSH6:NM_001281492:exon2:c.1129_1130del:p.R377fs, two-base deletion). This variant exhibited that two bases were missing in exon 2 of *MSH6* (Fig. 3A), which was originally composed of 1230 amino acids [24]. Owing to this variant, the protein was truncated and contained only 383 amino acids. This variant was likely pathogenic to the proband and has not been reported previously.

Next, Sanger sequencing was performed to verify the (c.1129_1130del, p.R377fs) *MSH6* variant in the other family members and provide genetic counseling based on genetic testing. The proband’s sister (II-3) and son (III-4) did not carry the germline variant in *MSH6*.

Table 1 presents the WES data for the proband (III-1) in family 2. The variants were filtered out based on their frequency, related function, and location. Based on the variant site sequencing and validation, a variant in *MLH1*, NM_000249.4:c.1731G > A, p.S577S, and rs63751657 (Fig. 3B) was identified at a splice site donor and considered a deleterious variant [25, 26]. Based on previous reports, we re-verified that the *MLH1* c.1731G > A, p.S577S variant is a pathogenic variant.

Sanger sequencing was then performed to verify the variant (c.1731G > A, p.S577S) in *MLH1* in other family members. The results revealed that the proband (III-1), his father (II-6), and son (IV-1) were all variant carriers, whereas his mother (II-7) was not.

### Analysis of mutant proteins using bioinformatic tools

**Fig. 4:**
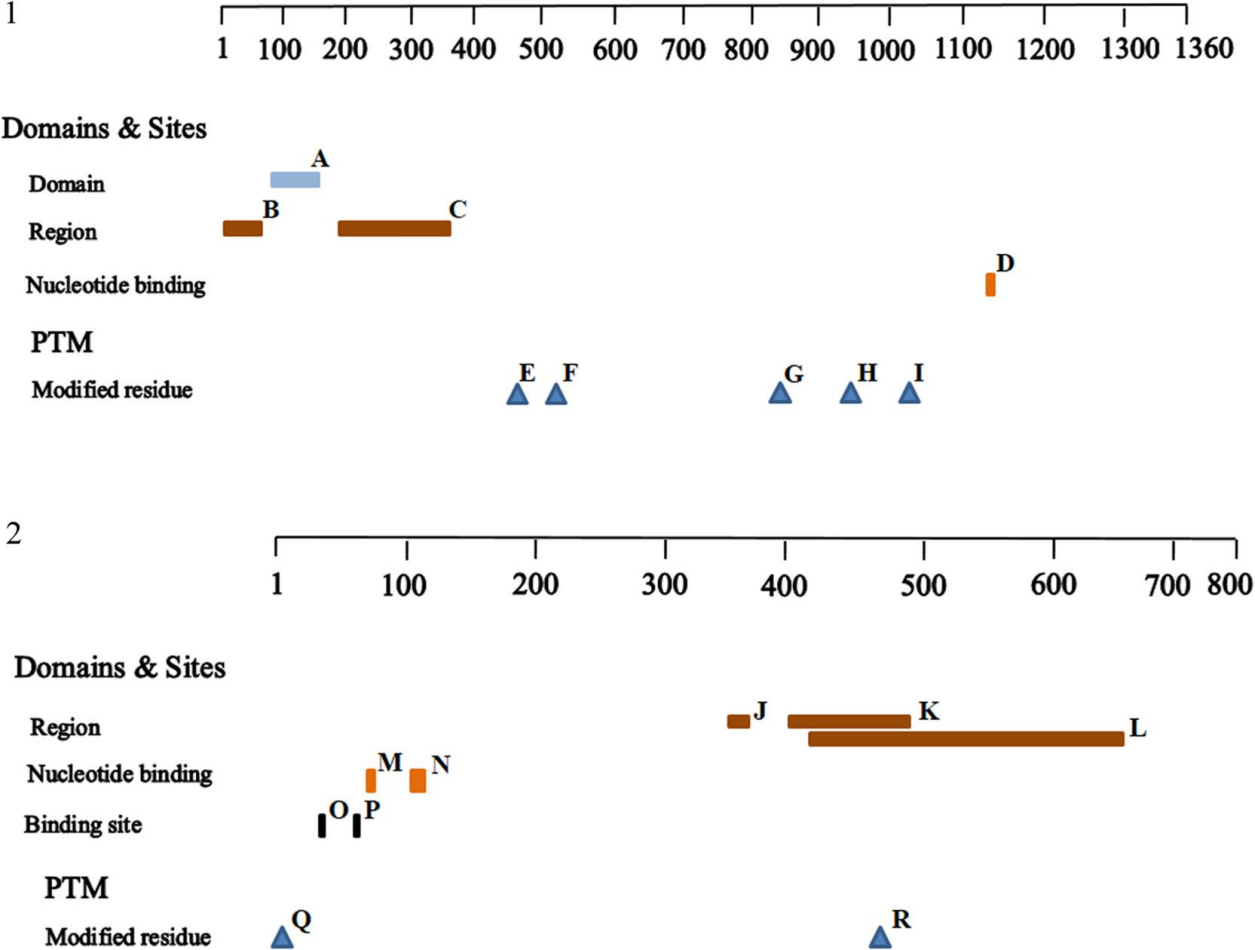
Mutant proteins analysis. (1) MSH6 protein analysis. **A** Region 92–154, PWWP domain; **B** Region 1–84, disordered; **C** Region 195–362, disordered; **D** NP_BIND 1134–1141, ATP; **E** MOD_RES 488–488, phosphothreonine; **F** MOD_RES 504–504, N6-acetyllysine; **G** MOD_RES 830–830, phosphoserine; **H** MOD_RES 935–935, phosphoserine; **I** MOD_RES 1010–1010, phosphothreonine. (2) MLH1 protein analysis. **J** Region 355–378, disordered; **K** Region 400–491, disordered; **L** Region 410–650, Interaction with EXO1; **M** NP_BIND 82–84, ATP; **N **NP_BIND 100–104, ATP; **O** BINGDING 38–38, ATP; **P** BINGDING 63–63, ATP; **Q** MOD_RES 2–2, N-acetylserine; **R** MOD_RES 477–477, phosphoserine

The *MSH6* (c.1129_1130del, p.R377fs) variant produced a truncated protein containing 383 amino acid residues. Sequence alignment and bioinformatic tools revealed that this led to a complete loss of the nucleotide ATP-binding region, which is found between residues 1134 and 1141 (Fig. 4D), and five post-translationally modified residues, including two phosphothreonine sites at residues 488 (Fig. 4E) and 1010 (Fig. 4I), one N6-acetyllysine site at residue 504 (Fig. 4F), and two phosphoserine sites at residues 830 (Fig. 4G) and 935 (Fig. 4H). However, no change was observed in the domain sites and the two disordered regions (Fig. 4A–C) between the wild-type and mutant proteins.

The variant (c.1731G > A, p.S577S) in *MLH1* was a deleterious variant. This variant caused skipping of the entire exon due to aberrant splicing in *MLH1*, resulting in a truncated protein (25; 26) that possibly affected the sequence after this variant site. Bioinformatics tools revealed that changes may occur in the two disordered regions at residues 355–378 (Fig. 4J) and residues 400–491 (Fig. 4K) and one phosphoserine-modified residue (residue 477, Fig. 4R), as well as in the region of contact with the EXO1 protein, spanning residues 410–650 (Fig. 4L). No differences were observed between the wild-type and mutant proteins in the two nucleotide ATP-binding locations at residues 82–84 (Fig. 4M) and residues 100–104 (Fig. 4N); two ATP-binding sites, one at residue 38 (Fig. 4O) and the other at residue 63 (Fig. 4P); and one N-acetylserine-modified residue (residue 2, Fig. 4Q).

## Discussion

*MSH6* does not account for a high proportion of LS-causing *MMR* variants [17, 27]. In this study, we identified a novel variant (*MSH6*:c.1129_1130del, p.R377fs) in *MSH6* and a known deleterious variant (*MLH1*:c.1731G > A, p.S577S) in *MLH1* within two typical families with LS. According to the ACMG classification, these two variants were considered likely pathogenic (PM2 + PM4 + PM6 + PP4) or confirmed pathogenic for LS.

No immunohistochemical staining of the MSH6 protein was observed in family 1, strongly suggesting that *MSH6* is the likely pathogenic variant. This variant truncated the MSH6 protein from its original 1230 amino acids to just 383 amino acids. The variant was located in the region of MSH6 and MSH2 interaction [24]. This variant likely reduces the DNA MMR ability, rendering the body more susceptible to tumors. Hence, we concluded that *MSH6* c.1129_1130del: p.R377fs is a likely pathogenic variant. Moreover, considering that both the proband’s father (I-1) and brother (II-2) died of CRC, the germline variant (c.1129_1130del, p.R377fs) in *MSH6* was speculated to be inherited from the proband’s father. In this family [1], although III-2 showed no signs of gastrointestinal tumors at the time of writing, we recommend that she continue to undergo regular gastroscopy examinations.

The proband with the *MSH6* gene variant was older and had a tumor recurrence, which is consistent with the results of previous reports [28, 29]. *MSH6* variants are observed in a relatively high proportion in elderly individuals, generally owing to their poor overall health. Patients with LS with an *MSH6* variant have a short survival period, accounting for the limited literature available on them. Furthermore, increasing evidence has shown that endometrial cancer is associated with *MSH6* variants [9, 30], possibly because the average life expectancy of women is higher than that of men. Moreover, female carriers of the *MSH6* variant are more likely to develop or cause these cancers owing to their reduced MMR function. Therefore, other male family members of patients with LS with *MSH6* variants, especially younger ones with no clinical manifestations, should be screened for the same variant sites. Once confirmed, continuous monitoring is necessary for early diagnosis and treatment, thereby prolonging the patient’s life.

In family 2, a known deleterious *MLH1* variant (c.1731G > A, p.S577S) was identified. Data from public databases revealed that the frequency of this variant site was low. Although this variant is a synonymous variant, existing literature has revealed that this variant is pathogenic. RT-PCR verified that this substitution resulted in the skipping of exon 15 and the mutant allele was not expressed in the full-length RNA. Furthermore, the transcript was modified to produce truncated proteins, which possibly affects the protein’s MMR function (25; 26). Based on the family pedigree [2] and genetic testing results, the variant was speculated to be inherited from the proband’s grandfather. However, IV-1 was only 4 years old at the time of writing. Hence, we recommend that he undergo regular gastroscopy examinations either when gastrointestinal symptoms manifest or after the age of 20 years.

Postoperative adjuvant therapy in advanced CRC usually involves the use of 5-fluorouracil (5-FU) alone or in combination with other agents [31]. However, patients with CRC with MSI rarely benefit from 5-FU-based adjuvant chemotherapy. Therefore, 5-FU is not recommended for chemotherapy in patients with MSI. Tumors with a deficient MMR repair are excellent targets for immunotherapy, particularly checkpoint inhibitors [32]. Programmed cell death 1 (PD-1), programmed cell apoptosis ligand 1 (PD-L1), and cytotoxic T lymphocyte antigen 4 (CTLA-4) are the most extensively studied checkpoint proteins that serve as coinhibitory regulators of T cell activation. Therefore, tumor patients with a deficient MMR or MSI-H are likely to benefit from anti-PD-1/PD-L1/CTLA-4 treatments [21, 33, 34].

This study contributes to the genotypic characterization of LS in China. Genotypic characterization is relevant for genetic counseling, diagnosis, and cancer prevention and treatment. WES is a quick, accurate, and reliable technique for identifying gene variants in patients suspected of having LS. WES has various applications in gene testing in tumors associated with LS. The highest lifetime risks of cancer are attributable to the presence of an *MLH1* or *MSH2* variant [35]. Therefore, the results of genotype–phenotype correlation studies would pave the way for tailoring more effective treatments for patients with LS.

The present study describes a novel variant in *MSH6* and a known *MLH1* variant in two unrelated Chinese families with LS. This study expands the spectrum of the known germline variants in *MMR* in the Chinese population. Although the presence of a novel variant in *MSH6* strongly indicates likely pathogenicity, additional evidence is essential to define its functional impact. This evidence may come from intra-familial variant segregation analysis, population frequency analysis, and in vivo/in vitro functional studies. Another limitation of this study is that some members of the two families could not be included because they were dead or were residing in other provinces of China. Genetic counseling and regular follow-ups should be conducted to provide individualized treatment for cancer-afflicted families with *MMR* expression deficiencies.

In conclusion, the novel frameshift variant (*MSH6*, c.1129_1130del, p.R377fs) is likely pathogenic for LS, and the variant (*MLH1*, c.1731G > A, p.S577S) is confirmed to be pathogenic for LS. It is essential to guide individualized treatment for families affected by cancer with *MMR* expression deficiency.

## Data Availability

The datasets used and analyzed during the current study are available from the corresponding author on reasonable request.
